# High-precision all-in-one dual robotic arm strategy in oral implant surgery

**DOI:** 10.1038/s41405-024-00231-6

**Published:** 2024-06-03

**Authors:** Gang Tang, Shibo Liu, Meng Sun, Yide Wang, Weidong Zhu, Dongmei Wang, Xiang Li, Hao Wu, Shaoyang Men, Liangbin Zhang, Changfen Feng, Yingfu Wang, Yuehua Ding

**Affiliations:** 1 Logistics Engineering College, Shanghai Maritime University, St. Haigang, Shanghai, 201306 Shanghai, China; 2 Electronic Information Engineering College, Nanjing University of Aeronautics and Astronautics, Nanjing, 210016 Jiangsu China; 3 Nanjing Panda Electronics Company Limited, Nanjing, 210000 China; 4 Institut d’Electronique et des Technologies du numerique, Polytech Nantes-Site de la Chantrerie, 44306 Nantes, France; 5 Department of Mechanical Engineering, University of Maryland Baltimore County, Baltimore, MD 21250 USA; 6 School of Mechanical Engineering, Shanghai Jiao Tong Univ, Shanghai, 200240 Shanghai, China; 7 Department of Gastroenterology, Changhai Hospital, Naval Military Medical University, Shanghai, 200433 China; 8 School of Medical Information Engineering, Guangzhou University of Chinese Medicine, postcode 510006 Guangzhou, China; 9 Scedent Medical Device Ltd., Shanghai, China; 10 Emergency Department of Stomatology, Suzhou Stomatology Hospital, Suzhou, Jiangsu 215031 China; 11 School of Electronic and Information Engineering, South China University of Technology, Guangzhou, 510640 China

**Keywords:** Dentistry, Dental equipment

## Abstract

**Introduction:**

Dental implantation has emerged as an efficient substitute for missing teeth, which is essential for restoring oral function and aesthetics. Compared to traditional denture repair approaches, dental implants offer better stability and sustainability. The position, angle, and depth of dental implants are crucial factors for their long-term success and necessitate high-precision operation and technical support.

**Method:**

We propose an integrated dual-arm high-precision oral implant surgery navigation positioning system and a corresponding control strategy. Compared with traditional implant robots, the integrated dual-arm design greatly shortens the preparation time before surgery and simplifies the operation process. We propose a novel control flow and module for the proposed structure, including an Occluded Target Tracking Module (OTTM) for occlusion tracking, a Planting Plan Development Module (PPDM) for generating implant plans, and a Path Formulation Module (PFM) for controlling the movement path of the two robot arms.

**Result:**

Under the coordinated control of the aforementioned modules, the robot achieved excellent accuracy in clinical trials. The average angular error and entry point error for five patients who underwent implant surgery using the proposed robot were 2.1° and 0.39 mm, respectively.

**Conclusion:**

In essence, our study introduces an integrated dual-arm high-precision navigation system for oral implant surgery, resolving issues like lengthy preoperative preparation and static surgical planning. Clinical results confirm its efficacy, emphasizing its accuracy and precision in guiding oral implant procedures.

## Introduction

Osseointegrated implants are cylindrical or screw-shaped fixtures made of biocompatible materials, precisely embedded into the alveolar ridge of the maxilla or mandible [1]. This approach expands the restoration options for partially and completely edentulous patients. It has been widely applied and researched in clinical settings [2–5]. With the rapid advancement of robotic technology, medical robotics [6] have emerged as a promising choice for osseointegrated implants. Medical robotics is a new interdisciplinary subject integrating medicine, mechanics, materials science, computer vision, computer graphics, robotics, and mathematical analysis. Extensive research efforts have enabled medical robots to be used in a variety of industries, including laparoscopic intestinal anastomosis [7, 8], tumor ablation [9], assistive wearable robots [10–12], and capsule robots [13, 14]. Robot-assisted surgery demonstrates numerous advantages over traditional techniques, including increased precision, efficiency, minimally invasive procedures, and enhanced safety. Consequently, it has become a focal point of research and a forefront trend [15].

Dental prostheses supported by osseointegrated implants are now considered the gold standard for replacing missing teeth [16], providing both functional and aesthetic benefits to patients. The key to the long-term success of these implants is accurate placement within the jawbone, including proper position, angle, and depth. To achieve greater precision, researchers are exploring the use of medical robots in oral implant placement [17–22].

The implant robot can achieve highly precise implant placement and positioning, thereby enhancing the stability and reliability of surgery while reducing associated risks. The accuracy of implant placement is one of the most important factors affecting the outcome of implant treatment and associated rehabilitation. A clinical study in reference [23] discussed potential influential factors and systematically summarized the accuracy of partially edentulous patient implantation with computer-guided surgery in recent years. Surgical navigation systems and template guidance met the high precision requirements for implant placement and positioning. However, surgeon’s vision and operation space are limited by the patient’s opening and the location of the missing tooth. Oral implant surgery encompasses two commonly used technologies: static guidance and dynamic navigation. Both technologies utilize computer assistance to achieve precise implant placement and positioning. Static guidance offers the advantages of simple operation and lower costs, making it particularly suitable for common implant surgeries. The drawbacks are also quite apparent. Due to the inability to make dynamic adjustments during surgery, it is susceptible to external factors such as patient head movement, potentially resulting in outcomes that deviate from expectations. In contrast, dynamic navigation systems offer higher precision and real-time adjustment capabilities, enabling the tracking of patient head and oral positions and the dynamic adjustment of surgical plans. Dynamic navigation proves advantageous, particularly in executing complex implant surgeries such as those involving unstable patient head positions or necessitating intricate implant positioning. However, its corresponding drawback is also notable, as dynamic navigation entails higher surgical costs. After analyzing past literature, it was found that a large number of studies emerged between 2004 and 2006. The studies performed by Chiu et al. [24], Kramer et al. [25], Brief et al. [26], and Casap et al. [27] indicated that the angle deviation between the implant axis and surgical plan of the dynamic navigation system was close to 4°. Afterward, a large number of researchers continued to study in this field and made progress based on previous research [28–35].

Robotic surgery offers significant advantages over traditional surgical methods, including sustained precision, improved stability, greater efficiency, and increased flexibility in performing implant preparation and placement. This advanced technology could eliminate the possible human error caused by physical fatigue and endurance limit. However, due to the high level of precision required for robotic surgery and the small margin for error, only a limited number of clinicians worldwide have employed it in their practice.

Boesecke et al. [17] first proposed robot-assisted dental implants and introduced a method with a preoperative 3D plan for inserting dental implants with an assisting medical robot. The study documented the placement of 48 implants in 16 clinical procedures using this approach and found that the gap between the top and tip of the implants was within 1–2 mm after CT fusion, pre- and post-operatively. The authors’ contribution is significant, as it lays the foundation for a new method of robot-assisted implant placement. However, there is limited information available in the literature regarding the specifics of the procedure and its execution. Sun et al. [18] provided a comprehensive and systematic overview of an automated robotic dental implant system. The system is equipped with a 6-degree-of-freedom (DOF) robot and a dental drill attachment that functions as a high-precision milling machine. This setup offers high accuracy in drilling implants with multiple roots, resulting in improved long-term stability and increased implant success rates. The coordinate measurement machine (CMM) mentioned therein requires a lot of preoperative preparation time for correction due to the separation from the drilling device.

In oral implant surgery, the use of image navigation technology is gaining momentum. Paper [19] introduces a stereo vision-based navigation system for a three-degree-of-freedom implant surgical assist robot, which was verified through experiments with a parallel-linked manipulator. Despite its promising results, this navigation system has a limitation in that it is susceptible to marker point occlusion caused by its fixed image capture device. Another notable development in this field is the Yomi-assisted dental surgery system developed by Neosis, based in Miami, Florida. Yomi received FDA approval in March 2017 [20]. The guidance system, known as haptic robotics, guides the surgeon through the drilling process based on the anticipated preoperative trajectory. The Yomi system offers a unique approach to implant surgery by providing physical guidance during the preparation of surgery. It limits the position, direction, and depth of the drill, eliminating the need for a customized surgical guide and reducing the risk of surgical deviation. With the capability to receive vibrational feedback, Yomi delivers unparalleled accuracy and predictability. However, it is important to note that the system should only be used under professional supervision and is very expensive.

In 2017, Zhao [21] made a major contribution to the field of dental implan- tology by launching the world’s first autonomous dental implant system. This intelligent robot is capable of performing surgical tasks directly on patients, with the ability to adjust itself continuously and automatically during the procedure, without any direct control from the surgeon. Although the concept of this technology is promising, there is limited research available to confirm its reliability and feasibility in clinical practice. Further clinical trials are needed toassess the accuracy of the robotic operations and the position of the implants.

To address the limitations in the field, we propose the High-precision All-in-one, Dual-Arm Robot for Oral Implant Surgery (HADAROIS) design featuring a dual robotic arm system. The upper arm holds the surgical tool, also known as the “oral implant hand-piece”, while the lower arm features a miniature multi-eye gaze positioning camera. This innovative design offers several structural advantages due to its high-precision parametric tracking capabilities. Firstly, the integration of the dual arms eliminates the need for preoperative calibration of the arm and optical positioning camera, significantly reducing preparation time. Secondly, the design simplifies the surgical procedure by eliminating the need to monitor and calibrate the surgical tools, reducing the chance of interruption. The patient only needs to wear a positioning marker that is tracked throughout the procedure, which minimizes the risk of obscuration. Finally, the proposed design allows for easy intraoperative adjustments, as the arm can be repositioned as needed to adjust the attitude of the optical positioning camera. This results in a more streamlined surgical process with increased accuracy, without the need for recalibration of the robotic arm and camera.

As our second contribution, we propose a cooperative control method to enhance the functionality of our proposed HADAROIS design. Our method includes a target tracking solution using image-guided techniques, which effectively resolves the challenge of surgical interruptions caused by obstructions such as the patient’s anatomy, medical devices, and organs during the procedure, ensuring its continuity. We also present a method for extracting CT image data to develop a precise implant plan and a more intuitive implant placement planning process by utilizing a device that displays the tissue at the target site. Furthermore, we outline the overall control process of HADORIS to enable a collaborative surgical procedure.

## Material and methods

### System design for HADAROIS

**Fig. 1:**
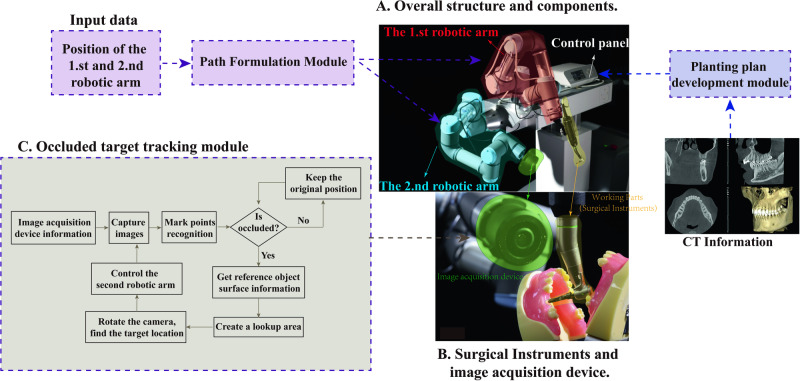
The proposed HADAROIS and its corresponding controlling modules. **A** The overall structure and module of the HADAROIS. **B** Surgical Instruments and image acquisition device. **C** The Occluded target tracking module.

In this section, we present the design and operation of the proposed HADAROIS system. Figure 1. illustrates the overall structure and components of the system, which consists of a support body, a control panel, two robotic arms, and an image acquisition device. The control panel and the two robotic arms are fixed to the support body, with the working parts for dental implants attached to the first robotic arm and the image acquisition device mounted on the second arm.

During the implantation surgery, it is necessary to obtain the real-time position of the patient’s head or oral cavity and register it in the virtual plan to determine the real-time posture of the first robotic arm’s endoscopic drilling tool during the surgery. The real-time acquisition of the implant position was achieved through visual localization and positioning markers. To describe the spatial position relationship of each component during the perforation preparation process, the coordinate system of the implantation plan is introduced. The implantation planning coordinate system is used to calibrate all the points involved in the operation that need to be identified in position and posture, in order to facilitate the digitization of the whole operation process. At the same time, it is convenient to plan the working path of the first manipulator.

**Fig. 2:**
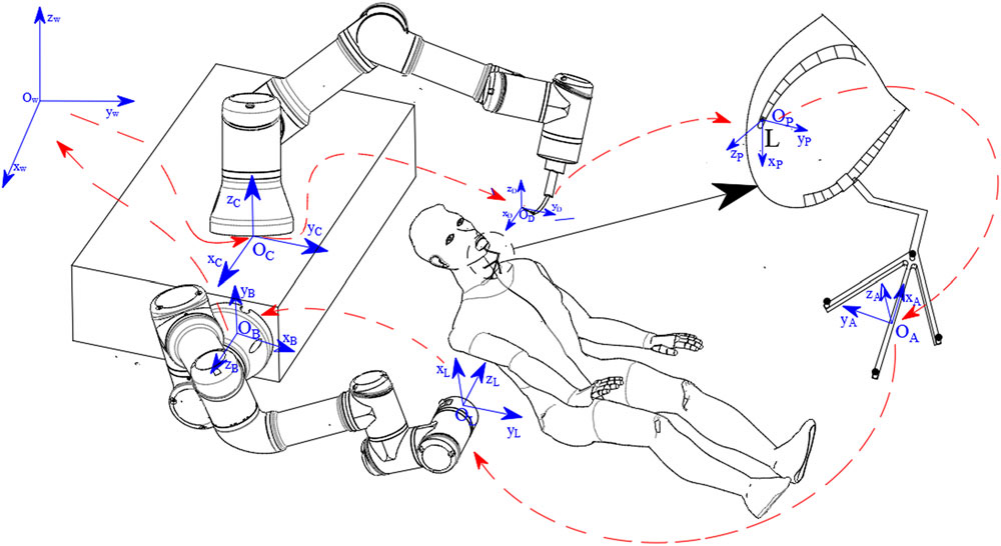
Coordinate system transformation diagram. O_w_ represents the global coordinate system, O_p_ represents the target position coordinate system, O_L_ represents the lens coordinate system, O_A_ represents the reference component coordinate system, O_B_ represents the second robot coordinate system, O_C_ represents the first robot coordinate system, and O_D_ represents the the tool coordinate system.

The accurate positioning of the robot end-effector is critical to the success of the implantation procedure. To achieve this, it is necessary to establish a series of coordinate systems for each involved component, including the global coordinate system ${O}_{w}-{x}_{w}{y}_{w}{z}_{w},$ the target position coordinate system of the implant ${O}_{p}-{x}_{p}{y}_{p}{z}_{p}$, the reference component coordinate system ${O}_{A}-{x}_{A}{y}_{A}{z}_{A}$, the lens coordinate system ${O}_{L}-{x}_{L}{y}_{L}{z}_{L}$, the second robot coordinate system ${O}_{B}-{x}_{B}{y}_{B}{z}_{B}$, the first robot coordinate system ${O}_{C}-{x}_{C}{y}_{C}{z}_{C}$, and the tool coordinate system ${O}_{D}-{x}_{D}{y}_{D}{z}_{D}$. The transformation relationships between these coordinate systems are presented in Fig. 2, illustrating their interdependence.

The image acquisition device is controlled by the second robotic arm, utilizing the Occluded Target Tracking Module (OTTM) to perform optical positioning and tracking of the target while identifying and avoiding any occlusions caused by the human body, medical devices, or organs. This effectively solves the problem of surgical interruptions during the procedure and ensures its continuity. The Planting Plan Development Module (PPDM) is used to extract CT image data and generate an efficient implant plan based on the information. The indication device, comprising a main body, a recognition body, and a data confirmation device, can be moved to display the tissue at the implant site on the interface, thereby making the placement planning more intuitive and convenient. The Path Formulation Module (PFM) plays a crucial role in the successful execution of the surgical procedure. It is responsible for determining the position, posture, and movement speed of the two robotic arms and coordinating their movements in real-time to ensure a seamless surgical experience. Furthermore, the PFM incorporates sophisticated algorithms for collision avoidance and interruption prevention, ensuring patient safety and procedural efficiency.

By reconstructing a virtual three-dimensional model of the patient’s mandible based on cone-beam computed tomography (CBCT) data, detailed anatomical information is obtained, allowing for precise determination of the number, size, position, and angle of the implants. Such meticulous planning ensures the optimal outcome of the implant surgery. The robot consists of a first robotic arm and a second robotic arm. The end effector of the first robotic arm is equipped with surgical tools, while the end effector of the second robotic arm is fitted with an optical capture and tracking device. Universal Robots 3 was selected as the robotic arm, and NDI’s Polaris Vicra was chosen as the optical capture and tracking device. In which, Universal Robots 3 manipulator adopts the products of Youao Robot Trading (Shanghai) Co., Ltd. The manipulator has six degrees of freedom of rotating joints, the payload is 3 kg, the working radius is 500 mm, and the repeatability is ±0.1 mm. NDL is the main 3D measurement system company in Canada, and its Polaris Vicra optical tracker has reliable measurement performance and small overall size. Its compact size allows medical equipment OEMs to integrate Polaris Vicra into a surgical workflow that requires smaller instruments and limited equipment or surgical space. The Polaris Vicra tracks 3D tool positions with submillimetre measurement accuracy and repeatability. The most subtle OEM surgical tool movements are precisely tracked and localized in real-time, with volumetric accuracy to 0.25 mm and 95% confidence interval of 0.5 mm. At the same time, Polaris Vicra has a smaller measurement volume optimized for targeted tool tracking within localized areas. Polaris Vicra tool geometries are correspondingly reduced, which allows for lighter and more ergonomic OEM instruments supporting very precise movements.

### Design of the image acquisition device

The HADAROIS system employs dynamic navigation to locate the dental appliance and capture the dental posture of the patient by using a close-range camera. This results in an increased input of data, leading to higher accuracy and faster response times. The vision tracking camera of the HADAROIS is positioned within a distance of 10 cm from the patient’s surgical area, which allows for better intraoperative control and minimizes the risk of system blindness due to accidental obstructions. The close proximity of the camera to the surgical area further enhances the accuracy of the system.

HADAROIS employs a novel, self-developed miniature camera to provide positioning and navigation functionality in its robotic system. The camera has a compact of 60 mm diameter and boasts an accuracy of 0.05 mm and a system refreshing rate exceeding 100 Hz, ensuring precision and real-time monitoring of the patient during the surgery. The Occluded Target Tracking Module (OTTM) is introduced for target point tracking, performing optical localization, target identification, and occlusion avoidance. The OTTM involves the calibration of the image acquisition component to determine internal parameters (e.g., focal length) and external parameters (e.g., position and orientation) for each camera. Subsequently, each camera in the image acquisition component takes simultaneous shots at a set frequency, and this process is repeated for each exposure.

Initially, multiple markers are designated for a target and the specified markers are detected. Subsequently, the center position of each marker on the target in the image captured by each camera is established and pinpointed. The three-dimensional spatial location of each marker’s center is also established, and the target’s center position is computed based on these center positions. When the number of identified markers on the target in the image captured by any camera falls below a predetermined threshold, it is determined that an occlusion has occurred.

The 3D position information of all the objects within the capture range of the image acquisition device is calculated and represented using point clouds on a grid to determine the object’s geometric boundaries. The Occluded Target Tracking Module (OTTM) then obtains a new image acquisition component position and orientation through the following steps:

1. The OTTM will create a three-dimensional area of regular space at a distance between the image acquisition component and the target center position as a finding area, the extent of which is determined by the reach of the mechanical device. Afterward, several sampling points are obtained on the lookup area.
2. The OTTM sets the center of the image acquisition device to these sampling points in turn and sets the video acquisition direction of the image acquisition component to a vector between the sampling points and the target center position.
3. Simultaneously rotate each camera along the video acquisition direction at the position of each sampling point. Taking successive exposure shots in the computer based on the internal and external parameters of the cameras until an intentional position is found such that all marker points of the target are acquired simultaneously by each camera.

After the intention position is obtained, the second mechanical wall control is finally controlled to move and rotate the image acquisition device to the calculated intention position.

### Planting Plan Development Module

The Planting Plan Development Module (PPDM) takes the patient’s CT scan as input and generates a corresponding implant plan.

**Fig. 3:**
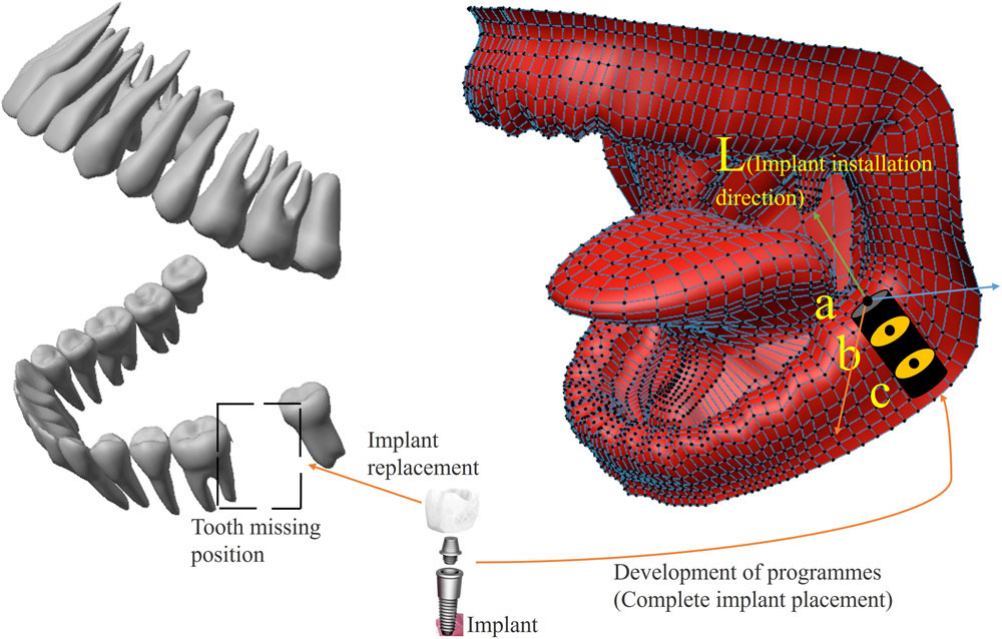
Schematic diagram of implantation position of the implant. The a is the boundary point between the air and gum, while the b and c are the boundary points between the gum and alveolar bone. L represents the direction in which the implant enters the gum and alveolar bone. The values of the a, b, and c are determined by CBCT measurements.

Firstly, CBCT images are acquired from patients wearing positioning and tracking pointing devices, and the image guidance system is configured. Secondly, the positioning ball located on the positioning and tracking pointing device is in a dominant state under the CBCT image, which facilitates the computer’s determination of the position and posture of the pointing device. Subsequently, CBCT image data of tissues, including gingiva and alveolar bone, are extracted and analyzed to identify the tissue types and the boundary information between them. This information is then used to formulate the optimal direction L of implants and the proposed planting scheme of the starting point and end point of the implantation. The specific generation method involves extracting the CBCT value of each point along the suggested implantation direction, using the position information of the current point on L as the horizontal axis and the corresponding CBCT image value as the vertical axis to establish a two-dimensional coordinate system. The difference between bone tissue and soft tissue leads to a significant disparity in the CBCT value between soft tissue and air, which is reflected in the image as a sudden change in the image gray value, and the fluctuation of the peak is displayed in the two-dimensional coordinate system. By using this established two-dimensional coordinate system, the boundary point a between air and gum, and the boundary points b and c between the gum and alveolar bone can be determined, and point b is chosen as the starting point of implantation. According to the pre-selected implant length, move the same length along the starting point b and L directions to find the end point of the implant, as shown in Fig. 3.

### All-in-one design

This section will introduce the integrated design of the camera input system and the execution robot arm. Currently, the major challenge facing robotic surgery is the extended duration of the operation, making it increasingly difficult to achieve the objective of reducing the surgeon’s operating time. The HADAROIS is designed to reduce the number of preoperative preparation steps and shorten the time of preoperative and intraoperative operations by means of a special structural design.

HADAROIS is designed with two robotic arms, the upper arm gripping the surgical tool and the lower arm with a miniature multi-eye gaze positioning camera. In order to make the two arms operate in an efficient and orderly manner, we propose the Path Formulation Module (PFM) control to implement the cooperative movement.

Under the control of the OTTM, the position and orientation of the tracked target can be obtained. The position and orientation of the working part (surgical tool) are determined in real-time based on the rotation angle of each joint on the first arm. The system translates the tracked target’s position and orientation from the coordinate system of the image acquisition device to that of the first arm using the relationship between the first and second arms. This enables the first arm to obtain the position and orientation of the tracked target in its own coordinate system. The specific position and direction of the predetermined path are thus obtained, and the first arm is guided to perform the task near the tracked target along the predetermined path. The computer continuously receives real-time information about the position and orientation of the working part and the first arm, and the image acquisition device on the second arm continuously adjusts its direction to capture the first arm and the working part. The system then calculates the minimum distance between the first arm, the working part, and surrounding objects based on the 3D information of the joint positions on the first arm and the surface points of the surroundings. It predicts any potential collisions between the first arm, the working part, and the surrounding objects and halts the movement of the first arm if necessary.

**Fig. 4:**
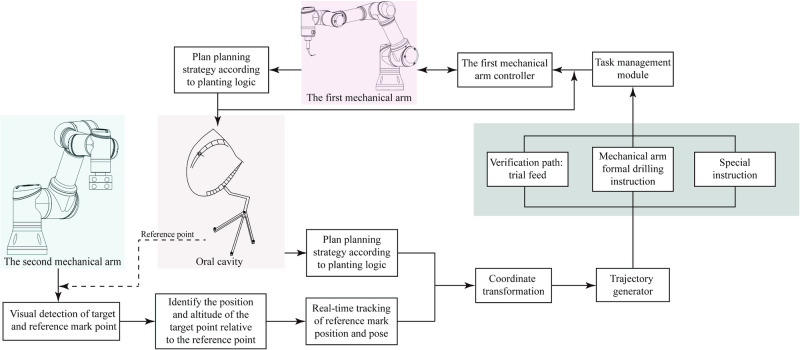
Control Strategy of cooperative operation of two manipulators. The second robot arm is equipped with a binocular camera to recognize the patient’s oral position and pose in real-time, facilitating the development of the implant strategy. After coordinate transformation, the trajectory generator completes the trajectory planning for the first manipulator. The task management module then issues motion instructions to the first manipulator.

The specific implementation method is shown in Fig. 4, and it can be divided into the following six steps:

1. Pre-operative preparation: The oral cavity of the subject is scanned by CBCT and a three-dimensional model of the oral cavity is obtained.
2. Target location determination: The optimal drilling location is determined by combining the target-indicating device with the three-dimensional oral cavity model. Thereafter, a reference device is installed at a proper position in the oral cavity, which is used to track and identify the patient’s head movement in real-time.
3. Pose recognition: Firstly, the binocular camera mounted at the end of the second mechanical arm is used to recognize the mark points on the target pointing device and the reference device, and at the same time, the automatic equipment is used to recognize the relative pose of the target point relative to the reference point. Secondly, the target-pointing device is removed, and the mark point on the reference device is tracked in real-time by the binocular camera to determine the posture of the patient’s head.
4. Coordinate transformation: Transforming the position and pose of the target point to the position and pose of the manipulator system. Trajectory planning of the first mechanical arm: inputting the pose of the target point into the trajectory planner to obtain the moving path in the joint space of the mechanical arm.
5. The first mechanical arm control module: Transmits the desired path of the mechanical arm to the control module, and calculates the torque and rotation speed of each joint drive device through the motion controller. End tool execution: after the first mechanical arm reaches the specified position, the end tool executes props to complete the final operation.
6. Attitude feedback: The feedback control is realized by detecting the rotation angle of each joint of the first manipulator in real time and comparing it with the expected value.

## Results

In this section, we outline the design and operational flow of the proposed HADAROIS system. We then present the results of our accuracy tests on oral implants, as well as a comparison between HADAROIS and expert surgeons in terms of complete oral implant procedures.

### Simulation experiments

**Fig. 5:**
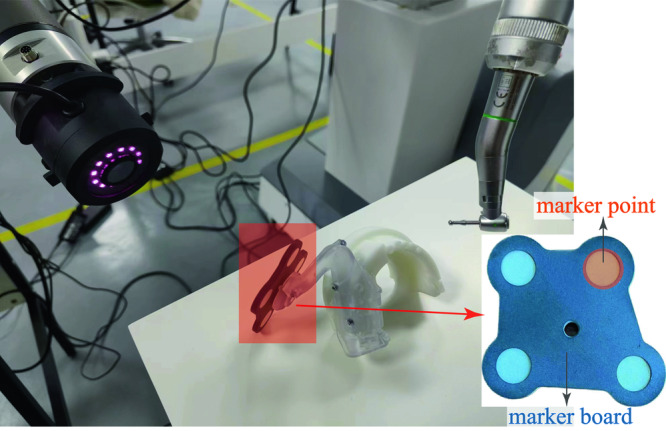
The illustration of the positioning device. The object within the orange frame is a mark board, consisting of four circular mark points. The mark board is secured to the subject’s teeth by a white bracket.

This section presents the simulation experiments of HADAROS, in order to demonstrate its feasibility for clinical surgeries. Two simulation experiments were conducted using 5 different dental models, and a total of 10 experiments were performed. The first step was to install a positioning device on the dental model, which consisting of a marker board with four markers and four spherical markers in predefined spatial positions. The marker structure is shown in Fig. 5, which displays the marker board and four spherical markers positioned at predetermined locations on the four corners of the main support.

**Fig. 6:**
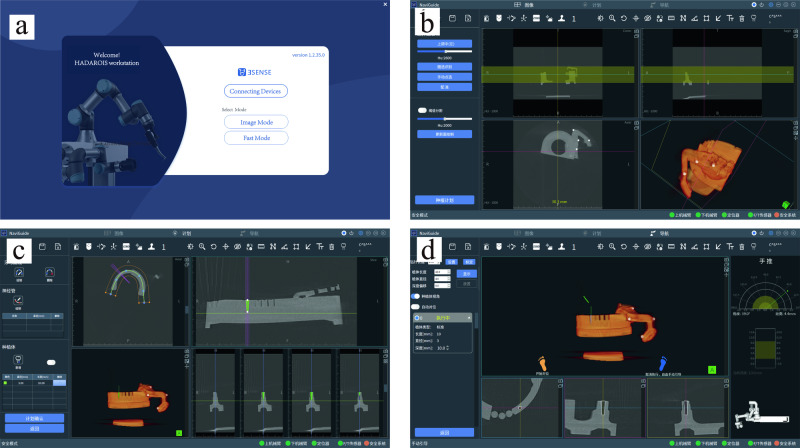
Screenshots of the graphical user interface in simulated experiments of oral implant surgery. **a** Upon opening the interactive interface, users are presented with the main interface including device connectivity options and the choice of two surgical modes (image mode and fast mode). **b** After completing the device connection, users enter the image mode interface, where the tooth model and the CBCT images of the positioning device are imported into the software. The four white dots in the image represent the position of the spherical markers in the CBCT, and selecting the area around them enables direct registration. **c** When the registration is complete, users can proceed with formulating the implantation plan. Once the dental arch shape is determined, which is shown in the upper left corner, the position, diameter, and depth of the dental implant fixtures can be selected. **d** Upon finalizing the surgical plan, the automatic implantation process can be initiated. The tilted green line in the image represents the real-time position of the drilling tool, allowing the lead surgeon to monitor the progress of the surgery and intervene manually when necessary.

The spatial relationship between the dental model and the positioning device was acquired using CBCT, and then four spherical markers were registered to the CBCT images. By utilizing the captured spherical marker points in the software, the registration process can be carried out to determine the spatial relationship between the entire marker structure and the tooth model. A screenshot of the interface is presented in Fig. 6. Subsequently, the video capture system positioned at the end of the second robotic arm can be employed to obtain the implementation coordinates of the oral cavity by capturing the marker points on the marker board.

After registration, the surgical path was planned in the software, including the direction, diameter, and depth of the drilling. When the planting plan was finalized, the robotic system proceeded to execute the drilling operation. The first robotic arm was locked to move only in a straight line in the direction of the planned angle. The drill tools were changed several times, and the drilling operation was repeated three to four times to complete the simulation surgery. The results were evaluated based on the angle error and entry point error. The angle error was defined as the difference between the planned drilling angle and the actual drilling angle, while the entry point error was defined as the difference between the planned entry point and the actual entry point. The average angular deviation is 1.54°e with a standard deviation of 0.67°, while the average entry-point deviation is 0.334 mm with a standard deviation of 0.202 mm. These results indicate that the robotic system can achieve accurate drilling during dental implant surgery. The average angle error and entry point error were both within an acceptable range, demonstrating that the robotic system can effectively guide the drilling operations. The small standard deviations of the angle error and entry point error indicate that the system has good consistency and repeatability.

The simulation experiment of the dental implant surgery robotic system has verified its ability to perform accurate drilling. The results have shown good consistency and repeatability of the system. The robotic system has the potential to become a valuable tool to assist dentists in implant surgery, and the clinical surgical results will be discussed in the following sections.

### Clinical end-to-end trials

**Fig. 7:**
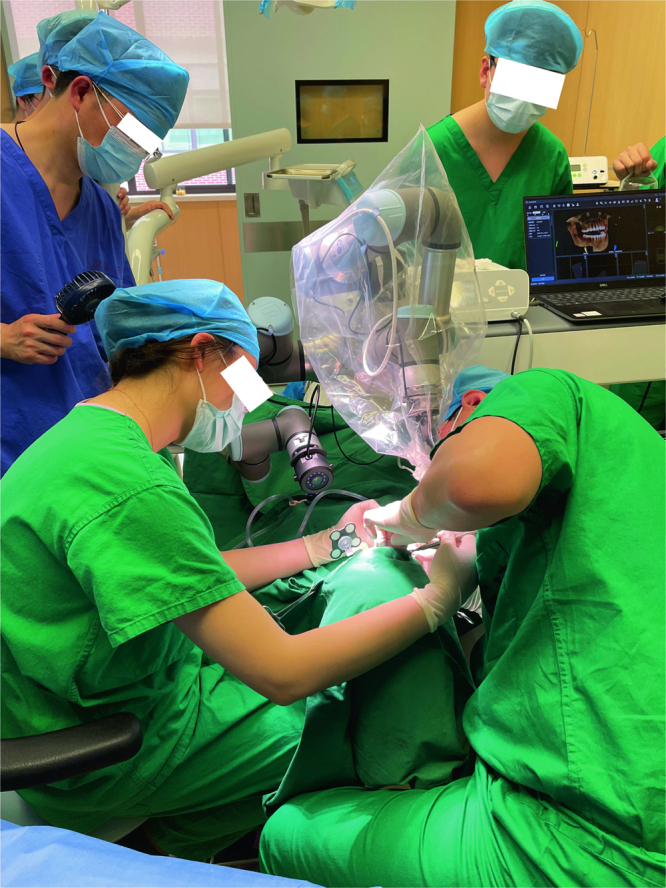
HADAR System Clinical Application Images. The dentist is performing a dental implant surgery using the HADAR System.

Finally, HADAR was utilized for performing oral implant surgeries on six patients, and Fig. 7 depicts clinical application images of HADAR system during implantation procedures. The Planting Plan Development Module (PPDM) was used to develop the surgical plan based on the CT information of the patients. The target position was then stabilized through the Occluded Target Tracking Module (OTTM) and the two robotic arms of HADAR were coordinated using the Path Formulation Module (PFM) to carry out the oral implant surgery.

**Fig. 8:**
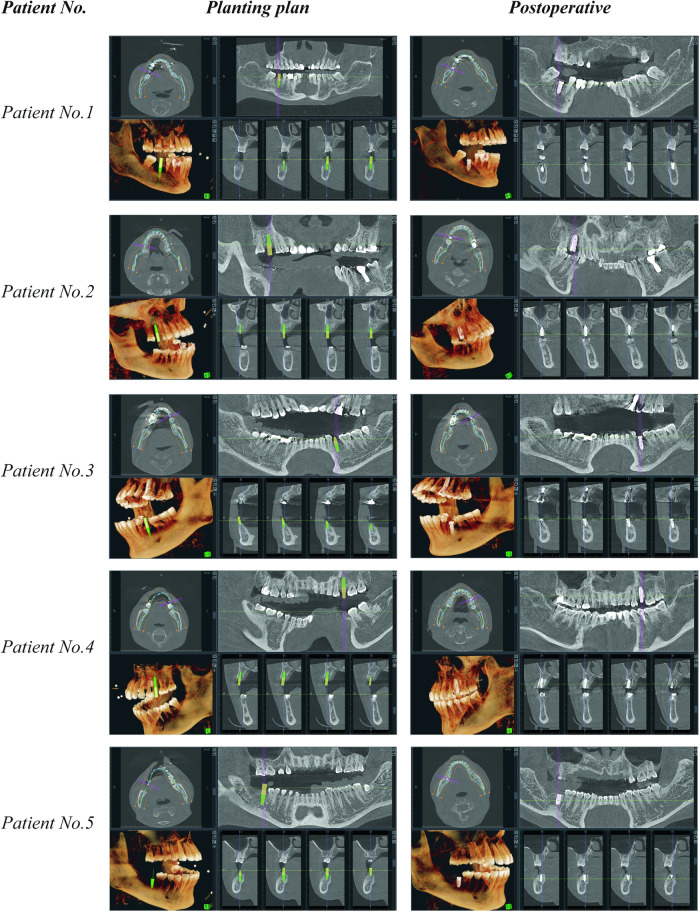
Implant plans and postoperative results of 5 patients. The left image shows the implant plan, and the right image shows the postoperative result.

**Fig. 9:**
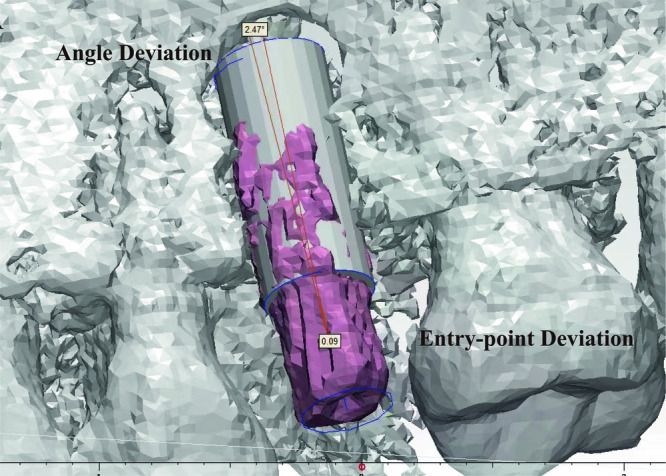
Angle deviation and entry point deviation visualization for postoperative analysis of patient No. 4. The white cylinder represents the planned implant position, while the irregular purple cylinder represents the actual implant position post-surgery. The distance between the starting points of the two positions is termed Entry-point Deviation, and the difference in their axes is termed Angle Deviation.

The results of the five subjects are shown in Table 1. Figure 8 showcases the surgical plans and postoperative outcomes of the five patients. The smooth cylinder in the figure shows the planned position of the implant in the surgical plan and the red solid shows the actual position of the implant in the patient. From the figure, it can be seen that the actual implant position of Patient No. 4 was 2.47° from the planned angular deviation and the entry point error was 0.09 mm. The angular deviation is the angle between the axis of the implant and the target axis in the surgical plan. The results of the clinical trials indicate that the average angular deviation of the surgical guide was 1.54° and the average entry-point deviation was 0.33 mm. These deviations are within the acceptable range for dental implant surgery Further analysis of the data reveals that Patient 1 had the smallest angular deviation of 0.96°, while Patient 4 had the largest angular deviation of 2.47°. In terms of entry-point deviation, Patient 4 had the smallest deviation of 0.09 mm, while Patient 2 had the largest deviation of 0.61 mm. The visualization of Patient No. 4 in the postoperative analysis of angular error and entry point error is shown in Fig. 9. Overall, the results demonstrate that the HADAROIS used in this study is a reliable tool for accurate implant placement in clinical practice, with deviations well within the clinically acceptable range.

**Table 1:** Control strategy of cooperate.

Subject No.	Angular deviation	Entry-point deviation
Patient 1	0.96°	0.26 mm
Patient 2	0.94°	0.61 mm
Patient 3	1.98°	0.46 mm
Patient 4	2.47°	0.09 mm
Patient 5	1.35°	0.25 mm

A postoperative comparison of the patient’s implant results with the surgical plan can be obtained as shown in Table 2. This analysis shows that while the deviation from the shoulder to the root increases with angular deviation, both deviations are still within the safe limit of 2 mm. In recent years, numerous studies have been conducted on implant placement accuracy [27–34], and the average accuracy results of these studies are summarized in Table 2. The angular deviation and entry-point deviation are listed for each study or subgroup, along with the year of publication. Our study shows the lowest angular deviation and entry-point deviation among all the studies listed.

**Table 2:** Mean implant accuracy results of this study in comparison with other published studies.

Study or subgroup	Angular deviation	Entry-point deviation	Year
Ersoy	4.25°	0.99 mm	2008
Cristache	2.46°	0.79 mm	2017
Schnutenhaus	4.46°	1.12 mm	2018
Derksen	2.73°	0.48 mm	2019
Chmielewski	4.89	1.6 mm	2019
Cassetta	2.63	0.8 mm	2020
Vinci	5	1 mm	2020
Scotty	2.56	0.95 mm	2021
Our Study	**1.54**°	**0.33** **mm**	2022

Our study achieves optimal values in angular deviation and entry-point deviation, which are 1.54º and 0.33 mm respectively, as highlighted in bold in the table.

## Discussion

In this study, we have proposed an integrated dual-arm oral implant surgery navigation positioning system that combines the mechanical arm with image capture equipment and the mechanical arm with surgical instruments into one unit. This design eliminates the need for traditional oral implant surgery navigation positioning system preoperative coordinate registration, greatly shortening the preparation time before surgery and reducing the overall surgical time.

The use of this new structure, in combination with the three proposed modules (PFM, PPDM, and OTTM), will make the surgical process more fluid, efficient, and safe. Through these modules, we have addressed several issues: (i) the problem of arm collision caused by the robot’s structural design, which is resolved by Module A, allowing both arms to operate efficiently and safely within the designated area; (ii) PPDM generates implant plans directly based on the patient’s CBCT images, providing suggestions to the main surgeon and reducing the impact of surgeon experience on the overall surgical outcome. This module also reduces the time needed for the manual selection of implant plans; (iii) OTTM addresses the issue of occlusion of positioning devices by surgical personnel during surgery. This enhances the robot’s robustness, allowing the surgery to proceed smoothly even when the user moves slightly or the image capture device is occluded.

We have demonstrated that the proposed structure and corresponding modules have already achieved the desired outcomes, reducing time, improving convenience, and achieving excellent surgical results. The robot has been introduced and put into use in hospitals, receiving positive feedback and excellent surgical outcomes. In future work, we will continue to improve the robot’s performance and address any issues that arise during surgery. Overall, this study provides a promising solution for improving dental implant procedures, making them more efficient, safe, and convenient for patients and surgeons alike.

## Conclusion

Taken as a whole, this study introduces an integrated dual-arm high-precision oral implant surgery navigation and positioning system, along with corresponding control strategies. This addresses issues encountered in traditional surgeries, such as lengthy preoperative preparation times and the inability to dynamically adjust surgical plans. Clinical application results demonstrate the effectiveness of the proposed method in conducting oral implant surgeries, exhibiting good accuracy and positioning capabilities. These findings highlight that our integrated system and control strategies can provide highly accurate navigation and positioning for oral implant surgeries.
