# Correction: High-precision all-in-one dual robotic arm strategy in oral implant surgery

**DOI:** 10.1038/s41405-024-00236-1

**Published:** 2024-06-13

**Authors:** Gang Tang, Shibo Liu, Meng Sun, Yide Wang, Weidong Zhu, Dongmei Wang, Xiang Li, Hao Wu, Shaoyang Men, Liangbin Zhang, Changfen Feng, Yingfu Wang, Yuehua Ding

**Affiliations:** 1 Logistics Engineering College, Shanghai Maritime University, St. Haigang, Shanghai, 201306 China; 2 Electronic Information Engineering College, Nanjing University of Aeronautics and Astronautics, Nanjing, 210016 Jiangsu China; 3 Nanjing Panda Electronics Company Limited, Nanjing, 210000 China; 4 Institut d’Electronique et des Technologies du numerique, Polytech Nantes-Site de la Chantrerie, 44306 Nantes, France; 5 Department of Mechanical Engineering, University of Maryland Baltimore County, Baltimore, MD 21250 USA; 6 School of Mechanical Engineering, Shanghai Jiao Tong University, Shanghai, 200240 China; 7 Department of Gastroenterology, Changhai Hospital, Naval Military Medical University, Shanghai, 200433 China; 8 School of Medical Information Engineering, Guangzhou University of Chinese Medicine, Guangzhou, postcode 510006 China; 9 Scedent Medical Device Ltd., Shanghai, China; 10 Emergency Department of Stomatology, Suzhou Stomatology Hospital, Suzhou, 215031 Jiangsu China; 11 School of Electronic and Information Engineering, South China University of Technology, Guangzhou, 510640 China

**Keywords:** Dentistry, Dental equipment

Correction to: *BDJ Open* 10.1038/s41405-024-00231-6, published online 03 June 2024

In this article the affiliation details for Weidong Zhu were incorrectly given as ‘Department of Mechanical Engineering, University of Maryland Baltimore Country, 200240 Baltimore, MD, USA’ but should have been ‘Department of Mechanical Engineering, University of Maryland Baltimore County, Baltimore, MD 21250, USA’.

In addition, the funding from ‘China Postdoctoral Science Foundation (grant number: 2023M732965)’ was omitted.

The original article has been corrected.

